# CT angiography predicts use of tertiary interventional services in acute ischemic stroke patients

**DOI:** 10.1186/1865-1380-4-62

**Published:** 2011-10-03

**Authors:** Lisa E Thomas, Joshua N Goldstein, Reza Hakimelahi, Yuchiao Chang, Albert J Yoo, Lee H Schwamm, R Gilberto Gonzalez

**Affiliations:** 1 Department of Emergency Medicine, Massachusetts General Hospital, Boston, MA, USA; 2 Department of Radiology, Massachusetts General Hospital, Boston, MA, USA; 3 Department of Medicine, Massachusetts General Hospital, Boston, MA, USA; 4 Department of Neurology, Massachusetts General Hospital, Boston, MA, USA

## Abstract

**Background:**

Patients with acute stroke are often transferred to tertiary care centers for advanced interventional services. We hypothesized that the presence of a proximal cerebral artery occlusion on CT angiography (CTA) is an independent predictor of the use of these services.

**Methods:**

We performed a historical cohort study of consecutive ischemic stroke patients presenting within 24 h of symptom onset to an academic emergency department who underwent emergent CTA. Use of tertiary care interventions including intra-arterial (IA) thrombolysis, mechanical clot retrieval, and neurosurgery were captured.

**Results:**

During the study period, 207/290 (71%) of patients with acute ischemic stroke underwent emergent CTA. Of the patients, 74/207 (36%) showed evidence of a proximal cerebral artery occlusion, and 22/207 (11%) underwent an interventional procedure. Those with proximal occlusions were more likely to receive a neurointervention (26% vs. 2%, *p *< 0.001). They were more likely to undergo IA thrombolysis (9% vs. 0%, *p *= 0.001) or a mechanical intervention (19% vs. 0%, *p *< 0.0001), but not more likely to undergo neurosurgery (5% vs. 2%, *p *= 0.2). After controlling for the initial NIH stroke scale (NIHSS) score, proximal occlusion remained an independent predictor of the use of neurointerventional services (OR 8.5, 95% CI 2.2-33). Evidence of proximal occlusion on CTA predicted use of neurointervention with sensitivity of 82% (95% CI 59-94%), specificity of 71% (95% CI 64%-77%), positive predictive value (PPV) of 25% (95% CI 16%-37%), and negative predictive value (NPV) of 97% (95% CI 92%-99%).

**Conclusion:**

Proximal cerebral artery occlusion on CTA predicts the need for advanced neurointerventional services.

## Background

Regional systems of care have been established in some localities, where acute ischemic stroke patients are preferentially admitted to "stroke centers" [1,2]. However, no formal guidelines exist for determining which patients should be transferred from a primary stroke center (PSC), capable of administering thrombolysis, to a comprehensive stroke center (CSC), with advanced services including endovascular capabilities. As a result, there can be tremendous heterogeneity in which patients remain at a PSC versus which are transferred to a CSC. Furthermore, many PSCs are likely capable of providing maximal management to stroke patients and may reserve transfer for those who need additional services available only at a CSC [3,4].

Efficient resource allocation may best be achieved by reserving such transfers for patients who will receive the most benefit. A rapidly available tool that predicts which patients are interventional candidates would help emergency physicians determine who might benefit from transfer to a CSC.

One candidate for such a tool is CT angiography (CTA), which can reliably detect large occlusive thrombi in proximal cerebral arteries [5]. While only 25-35% of patients with acute ischemic stroke have such occlusions, they are disproportionately responsible for high hospital costs, morbidity, and mortality [6,7]. As intravenous (IV) recombinant tissue plasminogen activator (rtPA) is less effective in recanalizing proximally occluded vessels [8], these individuals may preferentially benefit from advanced therapies at tertiary care centers. In particular, intra-arterial thrombolysis [9,10], mechanical clot disruption [11,12], and device-aided thrombus extraction [13-15] have been shown to recanalize occluded vessels at a rate higher than for IV rtPA, which may lead to better outcome [16]. Since multislice CT scanners are available 24/7 in the majority of US emergency departments [17], it may be that this technology can be harnessed to select patients for transfer.

We hypothesized that the presence of an occlusive thrombus in a proximal cerebral artery on CTA is an accurate predictor of the use of advanced neurointerventional services. We elected to perform an observational study at a center in which virtually all patients undergo emergency CT angiography as a clinical standard of care, in order to minimize selection bias.

## Methods

### Study design

This was an historical cohort study of consecutive ischemic stroke patients who presented to a single academic emergency department (ED) and who underwent emergent CTA. The study was approved by our Institutional Review Board.

### Setting and selection of participants

All patients presenting within 24 h of symptom onset in 2006 to the ED were prospectively captured as described [6]. This hospital is a Massachusetts Department of Public Health-certified Stroke Center and offers a full range of CSC capabilities including tertiary care interventional and neurosurgical services 24/7. Patients requiring such services were treated at our study hospital as needed without being transferred.

Although MR angiography (MRA) can also identify proximal vessel occlusion, we did not include these studies because MRI is not available in the emergency department at most hospitals [17] and is not a required emergent service for PSCs. However, 96% of hospitals can perform an emergency CT with contrast [17], and so likely have the ability to detect a proximal artery occlusion.

### Imaging

Standard imaging at our center for suspected acute ischemic stroke includes CTA and MRI. CT images were acquired according to standard protocols [6].

### Classification of proximal cerebral artery occlusion on CTA imaging

**Figure 1:**
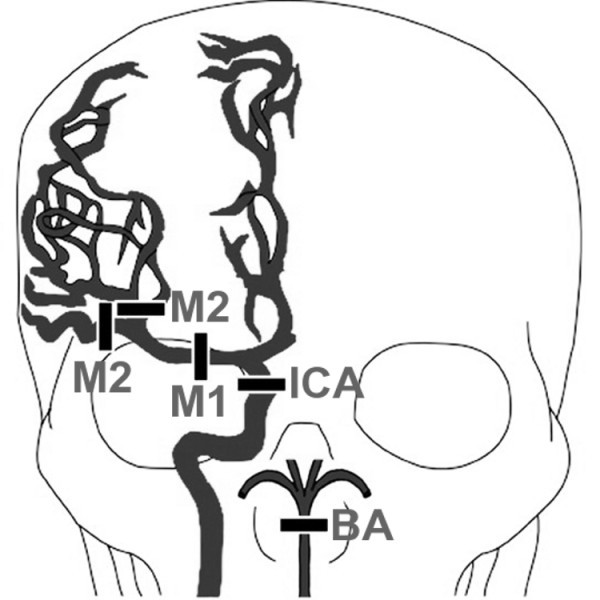
**Large vessel proximal cerebral occlusions**. The drawing depicts the major cerebral arteries and the sites of occlusion as specified by the Boston Acute Stroke Imaging Scale classification system (BASIS) [6]. Occlusion sites include the distal internal carotid artery (*ICA*), proximal segments of middle cerebral artery (*M1 *and *M2*), and the basilar artery (*BA*). Note the exclusion of other proximal arteries including the anterior cerebral, posterior cerebral, and vertebral arteries. The drawing is a modification of the illustration published [6].

**Figure 2:**
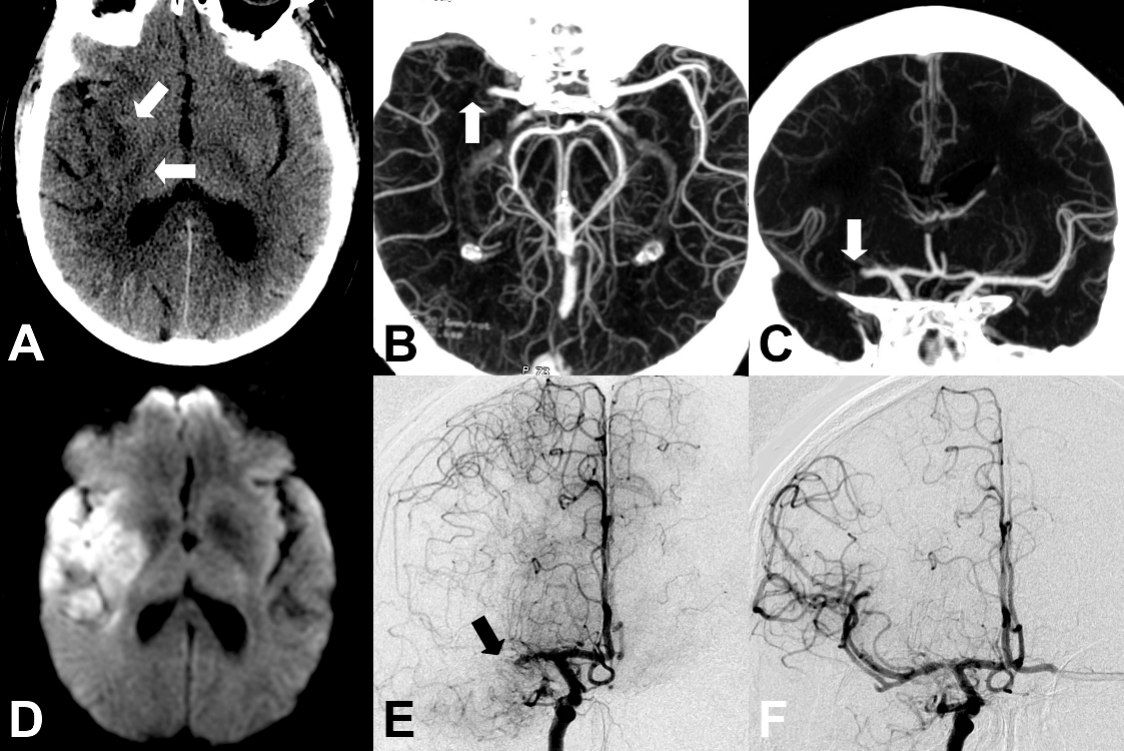
**Imaging of patient with proximal cerebral arterial occlusion**. Imaging of a 67-year-old male who presented 3 h after onset of left hemiparesis and aphasia with initial NIHSS of 18 and found to have proximal cerebral arterial occlusion is depicted here. After intra-arterial intervention, he was admitted to the neurosciences ICU, symptoms improved, and he was eventually discharged to a rehabilitation facility. (**a**) Noncontrast CT shows subtle hypodensity (*arrows*) in the right basal ganglia in right middle cerebral artery territory. (**b**) Axial CT angiogram reconstructed at the CT console immediately after the patient was scanned. The reconstruction was performed using the simple overlapping thick slab maximal intensity projection (*MIP*) algorithm and clearly shows (*arrow*) an occlusion of the proximal right middle cerebral artery (*M1 segment*). MIP parameters included 15-mm slab thickness overlapping at 3-mm intervals. (**c**) Coronal CT angiogram reconstructed at the CT console at the same time as **b **again demonstrates the right M1 artery occlusion (*arrow*). (**d**) MRI demonstrates the DWI hyperintense infarct in right MCA distribution. (**e**) Selective right internal carotid artery angiogram shows abrupt occlusion of blood flow at the right M1 segment (*arrow*) confirming CTA finding. (**f**) Post intra-arterial therapy angiography shows restoration of cerebral blood flow in the right middle cerebral artery and its branches.

Presence of a large-vessel proximal occlusive thrombus was defined as described previously [6]. This included obstruction in the distal/terminal (intracranial) internal carotid artery, proximal (M1 or M2) middle cerebral artery, and/or basilar artery (Figure 1). These regions were selected based on a prior study showing that occlusions of these segments were more likely to be associated with larger strokes [18] and based on the likelihood that proximal occlusions in these locations could be readily identified by physicians with minimal training in interpreting CTAs. The original neuroimaging report was reviewed by a neuroradiologist, who was blinded to whether the patient received any IA therapies, to confirm the official interpretations and to clarify any ambiguous descriptions to ensure uniform classification of proximal occlusion for study purposes. In the event of conflicting original and subsequent interpretations, a second neuroradiologist was available to review the images; however there was 100% interrater reliability with the original interpretation. An example of a patient with a proximal cerebral artery occlusion is shown in Figure 2.

### Outcome measures

The primary outcomes of interest were use of tertiary care neurointervention, including IA thrombolysis, mechanical clot retrieval or removal, or any neurosurgical procedure. We had 85% power to detect a 15% difference in the primary outcome between patients with and without proximal occlusion at the 0.05 level. Decision for the type of treatment used was based on clinical judgment of the treating cerebrovascular specialists. Secondary outcomes included need for ICU admission, length of stay, and disposition after hospital stay (categorized as discharge to home, transfer to a rehabilitation center/skilled nursing facility, or death).

### Data analysis

As most variables were not normally distributed, univariate analyses were performed using the Wilcoxon rank sum test for continuous variables and Fisher's exact test for categorical variables. Due to the small number of outcomes, we included proximal occlusion on CTA and only one additional variable, NIHSS score, in the multivariable logistic regression model. Goodness-of-fit test and regression diagnostics were performed for influential observations. Statistical analyses were performed using STATA software version 10 (STATACorp, College Station, TX).

## Results

During the study period, 290 patients who presented within 24 h of symptom onset were admitted with acute stroke. Of these, seven were excluded for enrollment in the DIAS-2 clinical trial [19] since the intervention was blinded. Another 76 were excluded for not having a CTA performed (61 had MRA for cerebrovascular imaging and 15 had no vessel imaging because of contraindications to both studies), leaving 207 patients for final analysis. The median time to registration in the ED from the time last seen well was 3.9 h (IQR 2-5.8 h). Thirty-three percent of patients presented within 3 h of symptom onset, 75% within 6 h and 90% within 12 h. Of this cohort, 25% of patients received IV rtPA, 2.4% received IA thrombolysis, 6.8% received a mechanical intervention, 3.3% underwent surgery (4 decompressive hemicraniectomies and 3 carotid endarterectomies), and 52% were admitted to the neuroscience ICU.

Table 1 shows patient characteristics among those receiving an advanced neurointervention. Of note, there was no significant difference in rate of IV rtPA use between those who did and did not receive an intervention. Table 2 shows the comparison of patients with and without proximal occlusion. In multivariable logistic regression, proximal occlusion on CTA was an independent predictor of the use of neurointerventional services after controlling for initial NIHSS score (Table 3). Finally, test characteristics for the ability of a proximal cerebral arterial occlusion to predict the need for neurointervention were calculated (Table 4).

**Table 1:** Characteristics of patients who received advanced neurointerventional procedures*

Characteristics	No neuro-intervention (*n *= 185)	Neuro-intervention* (n = 22)	p-value
Age (IQR)	74 (62-81)	80 (60-85)	0.2
Female	45%	32%	0.3
Transferred	45%	64%	0.1
Initial NIHSS (IQR)	7 (3-12)	20 (10-22)	0.0001
Time (h) to presentation (IQR)	4 (2-6)	3.6 (2.5-4.5)	0.2
Proximal occlusion on CTA	30%	86%	< 0.001
IV rtPA	24%	32%	0.4
Length of stay (days) (IQR)	5 (3-7)	8 (7-15)	< 0.001
Outcome:			0.007
Death	13%	27%	
Rehab	49%	64%	
Home	38%	9%	

*Neurointerventional procedures included intra-arterial thrombolysis, intra-arterial mechanical clot retrieval or manipulation, or any neurosurgical procedure.

IQR, interquartile range; SD, standard deviation.

**Table 2:** Comparing patients with and without proximal cerebral arterial occlusion on CTA

Characteristics	No proximal occlusion (*n *= 133)	Proximal occlusion (*n *= 74)	p-value
Age (IQR)	72 (60-80)	76 (68-83)	0.04
Female	46%	39%	0.4
Transferred	43%	54%	0.14
NIHSS (IQR)	4 (2-9)	17 (9-21)	0.0001
Time (h) to presentation (IQR)	4 (2.1-6)	3.8 (1.8-5.6)	0.3
IV rtPA	17%	38%	0.002
Length of stay (days) (IQR)	4 (3-7)	6 (4-10)	0.0001
Neuroscience ICU stay	35%	85%	< 0.0001
Any neurointervention	2%	26%	< 0.001
Neurosurgical intervention	2%	5%	0.2
IA thrombolysis	0%	9%	0.001
Mechanical IA procedure	0%	19%	< 0.0001
Outcome:			< 0.001
Death	6%	30%	
Rehab	45%	61%	
Home	49%	9%	

ICU, intensive care unit; IA, intra-arterial; IQR, interquartile range; SD, standard deviation.

**Table 3:** Predictors of need for any advanced neurointervention using multivariable analysis

Variable	OR (95% CI)	p-value
NIHSS (per unit increase)	1.1 (1.01-1.2)	0.03
Proximal cerebral artery occlusion	8.5 (2.2-33)	0.002

**Table 4:** Test characteristics of proximal cerebral artery occlusion on CTA predicting need for neurointervention

	Sensitivity (95% CI)	Specificity	PPV	NPV
Any neuro-intervention*	82% (59-94%)	71% (64-77%)	25% (16-37%)	97% (92-99%)

**IA thrombolysis**	86% (49-97%)	67% (66-67%)	8% (5-9%)	99% (97-99%)

**Mechanical IA procedure**	100% (79-100%)	70% (69-70%)	19% (15-19%)	100% (98-100%)

IA, intra-arterial; PPV, positive predictive value; NPV, negative predictive value.

*Any neurointervention includes IA thrombolysis, IA mechanical clot retrieval or manipulation, or any neurosurgical procedure.

## Discussion

We found that proximal cerebral artery occlusion on CTA predicts the use of acute neurointervention. While time to presentation and neurological exam findings are often used in decision-making regarding transfers, this specific radiographic finding appears to add independent value in predicting tertiary care interventions. Use of CTA in selected patients may therefore improve our ability to stratify which patients would benefit from emergent transfer to a CSC.

Although only a quarter of patients with a proximal occlusion actually received a neurointervention, distinguishing those with a large occlusion may be important for two reasons. First, if an occlusion is not seen, it is highly unlikely that a patient will need an intervention. In fact in our study, only 3% received an intervention without a large occlusion on CTA. All of these were patients with critical internal carotid stenosis that received carotid endarterectomies that were not performed on the same day as admission but during that hospital stay. Thus, most of the patients without proximal occlusion could potentially receive appropriate care at PSCs depending upon resources available. On the other hand, if a proximal occlusion is seen on CTA, these patients should be considered for emergent transfer or at least discussed with a CSC via teleradiology or phone consultation to determine whether they are interventional candidates. Even if they are not, they might still benefit from care at a CSC because they will tend to have larger strokes, worse outcomes [6], and may have more complicated care needs.

The commonly used practice of relying on clinical findings and noncontrast head CT for management decisions may provide inadequate information for triaging stroke patients for advanced therapies. For example, large artery occlusive strokes may not respond well to IV rtPA, but show better response to IA therapies [20,21]. In addition, vascular imaging provides independent information regarding the patient's prognosis [18]. As a result, current American Heart Association (AHA) guidelines endorse vascular imaging in the initial evaluation of the patient with acute ischemic stroke symptoms [22].

Our data confirm findings from others that patients with proximal occlusions tend to have higher NIHSS scores [23-26]. This raises the question of whether the NIHSS score alone can select those patients requiring advanced intervention. We conclude, however, that CTA does add independent value. First, one recent prospective study found that NIHSS alone has a poor negative predictive value for proximal occlusion amenable to intervention [27]. Second, we found that CTA provides independent information even when controlling for NIHSS. In particular, NIHSS is known to be influenced by location because it is so heavily weighted toward language function, with posterior circulation occlusions leading to a lower initial NIHSS but a worse clinical outcome [28,29].

The major limitation of our study design is that it is a single center retrospective cohort. We chose this design for our initial analysis because our center routinely performs CTA on almost all stroke patients, minimizing selection bias. However, patients presenting to an academic center with available tertiary care services may not reflect the full range of ischemic stroke patients that present to community hospitals. More than half of the patients that had proximal occlusions on CTA or received neurointervention were transferred from outside hospitals; this likely reflects a concentrating effect providing a population of more severe strokes than that which might present to any single community hospital. While this enriched our cohort for those that achieved the primary outcome, improving our statistical power, a multicenter study in a larger cohort will be necessary to validate these findings in a more representative population. There may be logistical, financial, and ethical considerations in consenting stroke patients for CTA in other practice settings where it is not routine, but our results appear to provide justification for such a larger, prospective study of the use of CTA to guide transfer decisions.

Another limitation was the exclusion of those who were unable to undergo CTA, most often due to IV contrast allergy and renal insufficiency. While many such patients would also be excluded from interventional neuroradiological procedures, it is possible that some would still have been candidates. Also, there is the possibility that CTA, performed at centers unaccustomed to acquiring it during acute stroke or at off hours, might perform an inadequate study that could delay treatment or transfer decisions, or preclude repetition of the study at the receiving facility.

Finally, the CTA findings were used in clinical decision making, potentially confounding our analysis. This likely overestimates the association of CTA proximal occlusion and neurointervention. Unfortunately, it would likely be unethical to "blind" clinical decision makers to CTA findings. In addition, our primary goal was to aid emergency physicians in predicting clinical options that would ultimately be offered to patients, and in a real world setting such decisions are expected to incorporate all available clinical and radiographic data.

Several factors must be considered prior to incorporating the use of emergency CTA in transfer decisions. AHA guidelines highlight that decision-making regarding IV thrombolytics should not be delayed for vascular imaging such as CTA, and protocols would need to be in place to ensure that treatment decisions for IV rtPA are made prior to initiation of further imaging [1,22]. Options can include only performing this test after IV thrombolysis in eligible patients, or only for those in whom decision-making would be changed based on the results. A rapid CTA can take less than 10 min to acquire, and the source images are available immediately on the CT scanner workstation. These images can then be rapidly processed and examined to detect proximal artery occlusion, and further studies should validate the ability of plain radiography technicians to generate the images and general radiologists or emergency physicians to reliably diagnose these occlusions. Another concern is the use of IV contrast, which can carry the risk of allergic reaction or contrast-induced nephropathy (CIN). Although traditionally thought to occur in 2-3% of cases, the risk of nephropathy after stroke or hospitalization is similar even without contrast, and many cases of CIN may simply be due to the nephropathy associated with hospitalization [30-36]. Finally, protocols should be in place to ensure that the study would not need to be repeated upon arrival to a tertiary care center, either due to an inadequate initial study or problems with image transfer between facilities. Prearranged transfer agreements, or even remote consultation via telephone or telemedicine [37], can ensure appropriate usage and communication.

## Conclusions

In summary, the finding of a large proximal cerebral arterial occlusion on CTA predicts the use of neurointerventional services in patients with acute ischemic stroke. Thus, our results provide justification for conducting future prospective studies on using CTA as a rapid decision-making tool to select patients who may be candidates for endovascular therapies at CSCs.

## Abbreviations

AHA: American Heart Association; CIN: contrast induced nephropathy; CSC: comprehensive stroke center; CTA: computed tomography angiography; ED: emergency department; IA: intra-arterial; ICU: intensive care unit; IV: intravenous; MRA: magnetic resonance angiography; MRI: magnetic resonance imaging; NIHSS: NIH stroke scale; PSC: primary stroke center; rtPA: recombinant tissue plasminogen activator

## Competing interests

The authors declare that they have no competing interests.

## Authors' contributions

LET gathered data, performed analyses, and drafted the manuscript. JNG performed statistical analyses, developed study design, and critically revised the manuscript for important intellectual content. RH gathered data and reviewed all imaging. AJY provided critical revision of the manuscript and figures for important intellectual content. LHS provided advice on analysis and critical revision of the manuscript for important intellectual content. RGG conceived the study, supervised data collection, and imaging analyses, and critically revised the manuscript for important intellectual content. All authors read and approved the manuscript.
